# The role of vocabulary concreteness in modulating embodied semantic representations in learners of Chinese as a foreign language

**DOI:** 10.3389/fpsyg.2026.1791237

**Published:** 2026-05-20

**Authors:** Zhiwei Cai, Lixia Wang, Jingchen Bu, Ning Fan

**Affiliations:** 1 College of Teacher Education, Baoding University, Baoding, Hebei, China; 2 College of International Exchange and Education, Hebei University, Baoding, Hebei, China; 3 College of Education, Hebei University, Baoding, Hebei, China

**Keywords:** abstract words, concrete words, embodied cognition, embodied semantic representation, learners of Chinese as a foreign language

## Abstract

Embodied cognition theory posits that semantic representations are instantiated through two primary mechanisms: perceptual–motor simulation and conceptual metaphor. To examine how lexical concreteness influences the embodied representation in learners of Chinese as a second language, two experiments were conducted. In Experiment 1, a spatial-action Stroop paradigm was employed using concrete and abstract verbs as stimuli. The results showed that only concrete verbs automatically elicited a front–back directional motion compatibility effect. In Experiment 2, concrete and abstract emotion words were used within a vertical space–valence compatibility paradigm. The findings revealed that both concrete and abstract emotion words exhibited a vertical spatial compatibility effect. Taken together, these results indicate that for learners of Chinese as a second language, the embodied representations of concrete foreign-language words are more diversified, whereas the embodied representation of abstract words is manifested primarily through spatial conceptual metaphors.

## 1 Introduction

Semantic representation refers to the mechanisms by which the brain encodes, stores, and processes meaning. It involves how individuals understand words, concepts, objects, and events, and how such understanding is organized and expressed within cognitive and neural systems (Markman and Dietrich, 2000). According to the degree to which the meanings expressed by words can be perceived in the real world—that is, their concreteness—words can be classified as concrete or abstract. Concrete words are processed faster and more accurately than abstract words in tasks involving semantic processing, such as lexical decision, word naming, and recall (Barber et al., 2013; Clark and Paivio, 1991). This performance advantage reflects differences in the representational mechanisms underlying concrete and abstract words.

To address the fundamental limitations of traditional cognitive theories based on abstract symbolic computation in explaining the origins of lexical meaning, embodied cognition theories emphasize the foundational role of the body in the cognitive system (Wu, 2019). Like other cognitive processes, language is grounded in bodily experiences derived from multiple sensory modalities or in experiences of the body itself (Lakoff and Johnson, 1999). The distinction between concrete and abstract words lies in the relative proportion and types of internal and external experiences and linguistic information associated with them. Sensorimotor information plays a dominant role in the representation of concrete words, whereas abstract words are broadly associated with emotional experience, spatial perception, and linguistic information (D’Angiulli et al., 2015; Vigliocco et al., 2014).

In empirical research on embodied semantic representation, concrete words are typically manifested through perceptual and motor simulation processes, whereas abstract words are more often reflected in metaphorical mappings between abstract concepts and perceptual experiences. However, findings regarding embodied semantic representation in second-language (L2) vocabulary remain inconsistent, and it is still unclear whether learners of Chinese as a foreign language differ in their embodied representations of concrete and abstract Chinese words. The present study focuses on learners of Chinese as a foreign language and employs two behavioral experiments to examine the effects of vocabulary concreteness on perceptual–motor simulation and vertical spatial conceptual metaphors. By exploring differences in embodied representation between concrete and abstract foreign-language vocabulary, this study aims to provide empirical evidence to inform and enhance vocabulary instruction in teaching Chinese as a foreign language.

From the perspective of embodied cognition, lexical meaning is jointly constructed from information across multiple dimensions (Wang et al., 2018; Xu et al., 2018). Broadly, this information can be divided into two categories: experiential information, including various forms of perceptual input, motor experience, and lived experience; and linguistic information, including syntax, morphology, and contextual cues (Bi, 2021; Ren et al., 2024). Empirical evidence suggests that concrete and abstract words rely on different mechanisms for semantic representation and comprehension, namely perceptual–motor simulation and conceptual metaphor, respectively.

Concrete words can be directly linked to perceptual experiences such as audition, vision, taste, touch, and olfaction, and their meanings are grounded in a perceptual symbol system based on the mental simulation of perceptual and motor experiences (Barsalou, 2010). For example, the verb “grasp” evokes a motor simulation of hand movement, illustrating how concrete action words engage perceptual–motor systems. In contrast, abstract words lack clear conceptual boundaries or referents that can be readily identified by the perceptual system (Fernandino et al., 2015) and are therefore primarily represented and understood through conceptual metaphors that map abstract meanings onto perceptual experience. By drawing on familiar and concrete experiences, abstract words construct unfamiliar and abstract concepts through metaphorical mappings, thereby achieving semantic representation (Lakoff and Johnson, 1980). For instance, emotion words such as “happiness” are often associated with an upward bodily orientation, reflecting the tendency to link positive affect to upward vertical space in perceptual–motor terms. These distinctions raise important questions about whether abstract words, rely solely on metaphorical mappings or can also engage perceptual–motor simulations similar to concrete words.

Researchers in bilingualism have also examined differences in embodied representational mechanisms between concrete and abstract L2 words within the framework of embodied cognition. Some studies have shown that abstract words do not rely solely on conceptual metaphors for their representation; rather, like concrete words, they can also elicit mental simulation of bodily actions and activate sensorimotor cortices, although the magnitude of activation is weaker than that observed for concrete words (Desai et al., 2013; Tian et al., 2020, 2023; Zhang et al., 2024). However, a study of Chinese–English bilinguals reported that English sentences containing abstract verbs failed to induce perceptual–motor simulation and did not produce an action–sentence compatibility effect (Wang et al., 2017). Thus, whether abstract words are directly grounded in sensorimotor experience and represented semantically via perceptual–motor simulation remains an open question.

Notably, the experimental paradigms used in previous studies typically required participants to engage in deep semantic comprehension and judgment. Consequently, the observed perceptual–motor simulation effects may be epiphenomenal, arising after semantic understanding has occurred (Meteyard et al., 2012), and therefore cannot establish perceptual–motor simulation as a prerequisite for the semantic representation and comprehension of abstract words. For this reason, experimental paradigms that more directly tap into the automatic processing of lexical semantics (e.g., the Stroop paradigm) are needed to provide a more fine-grained examination of perceptual–motor simulation in abstract words.

In line with this approach, several studies have employed the Stroop paradigm to investigate the automatic processing of abstract words, particularly emotion words, which constitute an important category of abstract vocabulary. Specifically, research has shown a close association between L2 emotion words and spatial perception, such that positive words tend to be mapped onto the spatial position “up,” whereas negative words are mapped onto “down” (Dudschig et al., 2014; Shen et al., 2014; Xie et al., 2014, 2015). For example, Dudschig et al. (2014) employed a spatial Stroop paradigm to examine whether L2 learners map positive and negative emotion words onto vertical spatial positions. In this paradigm, participants were required to press an upward or downward key in response to the color of the presented word. When the spatial association activated by the word (e.g., the positive word “happy” being associated with “up”) was congruent with the response direction (e.g., pressing the upward key), response times were facilitated; when the two were incongruent, responses were slowed. The results showed that, similar to native-language processing, positive emotion words in L2 facilitated upward responses, whereas negative emotion words facilitated downward responses. These findings suggest that spatial representations may be automatically activated during the processing of emotional meaning in L2.

Other research has further shown that the conceptual metaphors associated with abstract words are shaped by language and culture, and that L2 abstract words may exhibit spatial metaphorical orientations that differ from those in the native language (Shen et al., 2014; Wu, 2019). For example, Shen et al. (2014), using a spatial priming paradigm, found that both English emotion words in L2 and emotion words in Chinese as the native language exhibited vertical spatial metaphor effects. However, no spatial metaphor effect was observed along the horizontal dimension for the L2 English words. Similarly, Wu (2019) reported comparable findings: vertical spatial metaphor effects were observed for both Chinese emotion words in L2 and Malay emotion words in the native language, whereas along the horizontal dimension, only positive emotion words in the Malay native language showed a spatial metaphor effect. These findings suggest that while vertical spatial metaphors for emotional valence appear relatively robust across languages, horizontal metaphorical mappings may be more sensitive to language-specific and cultural influences.

However, most existing studies have not distinguished between different degrees of lexical concreteness, leaving it unclear whether concrete and abstract words are represented semantically through the same spatial conceptual metaphors. Given the uniqueness of the Chinese language and culture, investigating learners of Chinese as a foreign language allows, on the one hand, a direct comparison between concrete and abstract words in terms of their spatial conceptual metaphors, and on the other hand, an examination of whether Chinese culture shows distinctive characteristics in the spatial metaphorical representation of lexical semantics.

At present, there is no consensus on how foreign language learners embody the semantic representations of L2 words, and no study has specifically examined whether learners of Chinese as a foreign language process concrete and abstract Chinese words through the same embodied representational mechanisms. Therefore, the present study recruited international students learning Chinese in China to investigate the characteristics of lexical semantic representation to address the following questions:

(1) For learners of Chinese as a foreign language, is lexical semantic representation also embodied through perceptual–motor simulation and conceptual metaphor?
(2) Do concrete and abstract words show differential reliance on these two embodied representational mechanisms?

The study comprised two behavioral experiments, employing a spatial action Stroop paradigm with verbs (Dudschig et al., 2014) and a vertical space–valence congruency paradigm with emotion words (Shen et al., 2014; Xie et al., 2014). Reaction times served as dependent measure to examine the effects of lexical concreteness on perceptual–motor simulation and spatial conceptual metaphor in foreign-language lexical processing. Specifically, the spatial action Stroop paradigm was used to test whether the conceptual representation of foreign-language verbs involves automatic perceptual–motor simulation, whereas the vertical space–valence congruency paradigm assessed whether the semantic representation of foreign-language emotion words can automatically activate valence-related vertical spatial perception. Based on prior research, we hypothesized that learners of Chinese as a foreign language would activate perceptual–motor simulations when comprehending both concrete and abstract concepts, producing congruency effects along both horizontal and vertical dimensions.

## 2 Experiment 1: influence of concreteness on perceptual–motor simulation of verbs

### 2.1 Participants and materials

Twenty-four international students learning Chinese in China were recruited as participants (6 males, 18 females; mean age = 25.45 years, SD = 3.12). All participants were native speakers of Vietnamese and had begun learning Chinese after the age of 18. Their mean length of Chinese learning was 3.17 years (SD = 1.26), and all had passed Hanyu Shuiping Kaoshi (HSK) Level 4 within the past 6 months. Participants self-rated their Chinese listening, speaking, reading, and writing proficiency using a 5-point Likert scale (1 = very unproficient, 5 = very proficient), yielding mean scores of 3.91 (SD = 0.57), 3.62 (SD = 0.56), 3.70 (SD = 0.61), and 3.25 (SD = 0.87), respectively. All participants had normal or corrected-to-normal vision and received modest monetary compensation after completing the experiment. The study was approved by the Ethics Committee of Affiliated Hospital of Hebei University (protocol code: HDFY-LL-2021-178). All participants provided written informed consent prior to the experiment in accordance with the Declaration of Helsinki. The same ethical procedure was applied to the subsequent experiments.

A total of 201 two-character Chinese verbs at or below HSK Level 6 were selected from the New HSK Syllabus. These items constituted an initial candidate pool and were subjected to a series of subjective linguistic evaluations. Familiarity ratings were collected from 22 international university students who did not participate in the formal experiment (all with HSK Level 4 or above). Familiarity was rated on a 5-point scale (1 = very unfamiliar, 5 = very familiar; 0 = not recognized). Candidate items were excluded if more than 30% of raters reported not recognizing the word or if the standard deviation of familiarity ratings exceeded 3.0. This screening procedure yielded 169 verbs with mean familiarity ratings above 3.50, which formed the final candidate pool. In addition, 26 native Chinese university students rated these verbs for concreteness (1 = very concrete, 7 = very abstract), directionality (1 = strongly toward the body, 7 = strongly away from the body), and emotional valence (1 = very negative, 9 = very positive). The average intraclass correlation coefficients (ICCs) across raters were 0.72 for familiarity, 0.71 for concreteness, 0.75 for directionality, and 0.70 for emotional valence.

Based on the overall distribution of ratings and with the aim of selecting words that clearly represented the two ends of the concreteness continuum while retaining a sufficient number of stimuli, verbs with concreteness scores below 3.2 were classified as concrete, whereas those with scores above 4.8 were classified as abstract. Verbs with directionality scores below 3.2 were classified as toward verbs (describing actions toward one’s own body), whereas those with scores above 4.8 were classified as away verbs (describing actions away from the body).

The final stimulus set comprised 16 verbs in each of four categories: concrete toward verbs (e.g., 抓取/grasp), concrete away verbs (e.g., 发球/serve), abstract toward verbs (e.g., 吸收/absorb), and abstract away verbs (e.g., 传播/disseminate). One-way analyses of variance confirmed that the four verb groups were matched on emotional valence, familiarity, and number of strokes. Detailed stimulus characteristics are reported in Table 1.

**Table 1:** Matching results for the verb stimuli used in Experiment 1 (*M* ± *SD*).

Word type		Concreteness	Directionality	Emotional valence	Familiarity	Number of strokes
Concrete	Toward verbs	2.17 ± 0.43	2.37 ± 0.38	5.07 ± 0.20	4.27 ± 0.56	16.87 ± 4.04
	Away verbs	2.08 ± 0.28	5.42 ± 0.44	5.05 ± 0.55	4.33 ± 0.51	16.75 ± 3.78
Abstract	Toward verbs	5.20 ± 0.26	2.30 ± 0.32	5.12 ± 0.52	4.48 ± 0.38	16.68 ± 3.59
	Away verbs	5.12 ± 0.25	5.25 ± 0.34	5.21 ± 1.02	4.45 ± 0.31	16.50 ± 5.29

### 2.2 Procedure

The experiment employed a 2 (concreteness: concrete vs. abstract) × 2 (directionality: toward vs. away) × 2 (response direction: toward vs. away) fully within-subjects design. Reaction time (RT) and accuracy in a color judgment task served as the dependent variables.

The experimental program was implemented using E-Prime 3.0 and run on a Dell desktop computer equipped with a 20-inch color LED monitor (resolution: 1920 × 1,080; refresh rate: 60 Hz). The viewing distance was approximately 70 cm. Following the procedure of Dudschig et al. (2014), the keyboard was rotated 90° counterclockwise and positioned vertically. Participants placed their left and right index fingers on the F and H keys, respectively. They were instructed that when a word appeared in a warm color [red: RGB (255, 0, 0); orange: RGB (255, 128, 0)], they should lift their left index finger from the F key and retract their arm to press the A key, corresponding to a toward-the-body response. When a word appeared in a cool color [blue: RGB (0, 0, 255); green: RGB (0, 192, 0)], they should lift their right index finger from the H key and extend their arm to press the L key, corresponding to an away-from-the-body response. The mapping between color category (warm vs. cool) and response direction (toward vs. away) was counterbalanced across participants.

Each trial began with the presentation of a white fixation cross (“+”) at the center of a black screen for 750 ms, followed by the target word. Participants were required to judge the color of the word by pressing the corresponding key within a response window of 1,500 ms. The stimulus disappeared immediately after a response was made.

The experiment consisted of two blocks, each containing 68 trials. The first four trials in each block served as practice trials and were excluded from analysis. The remaining 64 experimental trials comprised concrete toward verbs, concrete away verbs, abstract toward verbs, and abstract away verbs, with 16 items in each category, presented in a randomized order. The response direction assigned to each word was reversed between the two blocks. The two colors corresponding to the same response direction were counterbalanced across participants. Participants first completed a practice session to familiarize themselves with the task and response mapping before proceeding to the formal experiment, which lasted approximately 8 min.

### 2.3 Results and analysis

Trials with incorrect responses and reaction times (RTs) shorter than 200 ms were excluded. Extreme values were further removed using a ± 3 SD criterion, resulting in the exclusion of less than 5% of the total data. Descriptive statistics for each condition are presented in Table 2. Reaction time was analyzed using linear mixed-effects models (LMMs). Each model included the three experimental factors—Concreteness (concrete vs. abstract), Directionality (toward vs. away), and Response Direction (toward vs. away)—as fixed effects, as well as all possible interactions. Random intercepts were included for both participants and items, and random slopes for each within-subject factor were incorporated for participants when model convergence allowed. This approach accounts simultaneously for variability across participants and items.

**Table 2:** Mean reaction times and accuracy for the lexical color judgment task in Experiment 1 (*M* ± SD).

Concreteness	Directionality	RTs (ms)		ACC (%)	
		Toward response	Away response	Toward response	Away response
Concrete	Toward verbs	818 ± 146	806 ± 146	99.50 ± 2.45	98.49 ± 2.67
	Away verbs	845 ± 153	787 ± 120	98.00 ± 3.82	98.50 ± 3.19
Abstract	Toward verbs	834 ± 139	798 ± 134	98.73 ± 3.13	98.75 ± 3.05
	Away verbs	821 ± 135	808 ± 131	98.99 ± 2.31	99.25 ± 2.03

The linear mixed-effects model revealed substantial variability in reaction times across participants, with the variance of participant intercepts estimated at 21405 (SD = 146). In contrast, the variance attributable to items was effectively zero, indicating negligible item-level variability. The residual variance was 24,477 (SD = 156), suggesting that while individual differences contributed to overall variability, the largest portion of variance remained unexplained by the model.

Analysis of the fixed effects revealed that reaction times were significantly affected by Directionality, with responses being slower for verbs indicating away movement compared to toward [*β* = 0.13, *t* = 2.31, *p* = 0.021, 95%*CI* = (0.019, 0.240)]. The two-way interaction between Concreteness and Directionality was significant [*β* = −0.206, *t* = −2.60, *p* = 0.009, 95%*CI* = (−0.361, −0.051)], as was the interaction between Directionality and Response Direction [*β* = −0.249, *t* = −3.16, *p* = 0.002, 95%*CI* = (−0.404, −0.095)]. Furthermore, the three-way interaction among Concreteness, Directionality, and Response Direction was significant [*β* = 0.361, *t* = 3.23, *p* = 0.001, 95%*CI* = (0.142, 0.580)], indicating that the effect of one factor depended on the combination of the other two. No other main effects or two-way interactions reached conventional significance levels (all *ps* > 0.09).

To further investigate the significant three-way interaction, estimated marginal means (EMMs) and pairwise comparisons were computed, with *p*-values adjusted for multiple comparisons using the Tukey correction. For trials with responses toward, concrete verbs showed significantly faster reaction times for toward-directed verbs compared with away-directed verbs (*t* = −2.305, *p* = 0.023), whereas abstract verbs did not show a significant difference (*t* = 1.343, *p* = 0.181). For trials with responses away, concrete verbs again showed a significant effect of verb direction, with faster responses to away-directed verbs (*t* = 2.116, *p* = 0.036), whereas abstract verbs showed no significant difference (*t* = −0.633, *p* = 0.528).

The pattern of the three-way interaction and simple effects indicates that the interaction between verb directionality and response direction emerged only for concrete verbs. No Stroop-like effect related to bodily movement direction was observed for abstract verbs.

## 3 Experiment 2: effects of concreteness on vertical spatial conceptual metaphors of emotion words

### 3.1 Participants and materials

Twenty-four international students learning Chinese in China participated in the experiment (4 males, 20 females; mean age = 25.83 years, SD = 2.67). All participants were native speakers of Vietnamese and had begun learning Chinese after the age of 18. Their mean length of Chinese study was 2.96 years (SD = 1.41), and all had passed HSK Level 4 within the past 6 months. Participants self-rated their Chinese listening, speaking, reading, and writing proficiency on a 5-point scale, yielding mean scores of 3.83 (SD = 0.55), 3.41 (SD = 0.64), 3.83 (SD = 0.55), and 3.13 (SD = 0.78), respectively. All participants had normal or corrected-to-normal vision and received modest monetary compensation upon completion of the experiment.

A total of 658 emotion words at or below HSK Level 6 were selected from the Chinese Affective Words System (Wang et al., 2008). Following the same rating procedure and standards as in Experiment 1, an additional 22 international students with HSK Level 4 or above rated the familiarity of these words on a 5-point scale. Words with mean familiarity ratings above 3.50 were retained, resulting in 542 candidate emotion words.

Next, 26 native Chinese university students rated these candidate words for concreteness on a 7-point scale. One-way analyses of variance were used to match the four emotion-word groups on valence, arousal, familiarity, and total number of strokes. Emotion words with valence ratings above 6.0 were classified as positive, and those below 4.0 as negative. Words with concreteness ratings above 4.8 were classified as abstract, and those below 3.2 as concrete.

The final stimulus set comprised 80 emotion words, with 20 items in each of four categories: concrete positive words (e.g., 微笑/smile), concrete negative words (e.g., 灾害/disaster), abstract positive words (e.g., 风趣/wittiness), and abstract negative words (e.g., 嫉妒/jealousy). Detailed stimulus characteristics are presented in Table 3.

**Table 3:** Matching results for the emotion word stimuli used in Experiment 2 (*M* ± *SD*).

Word type		Concreteness	Emotional valence	Arousal	Familiarity	Number of strokes
Concrete	Positive	2.38 ± 0.49	7.02 ± 0.38	5.97 ± 0.72	4.70 ± 0.22	16.60 ± 5.20
	Negative	2.62 ± 0.45	3.03 ± 0.37	5.95 ± 0.62	4.57 ± 0.29	16.65 ± 4.34
Abstract	Positive	5.42 ± 0.38	7.18 ± 0.30	6.27 ± 0.48	4.58 ± 0.29	16.80 ± 4.67
	Negative	5.24 ± 0.37	3.14 ± 0.43	6.07 ± 0.54	4.52 ± 0.23	17.10 ± 3.75

### 3.2 Procedure

The experiment employed a 2 (concreteness: concrete vs. abstract) × 2 (emotional valence: positive vs. negative) × 2 (dot location: top vs. bottom) fully within-subjects design. Reaction time (RT) and accuracy in a spatial dot detection task served as the dependent variables.

The experimental setup was identical to that of Experiment 1. The classic vertical spatial–valence congruency paradigm was adopted (Shen et al., 2014; Xie et al., 2014). Each trial began with the presentation of a white fixation cross (“+”) at the center of a black screen for 500 ms, followed by a white emotion word presented centrally for 3,000 ms. Participants were instructed to judge whether the word was positive or negative. After a 500 ms blank screen, two white squares (1.3 cm × 1.3 cm) of identical size appeared simultaneously at the top and bottom of the screen for 250 ms. Following a 500 ms blank interval, a white dot (5 mm in diameter) appeared randomly in either the top or bottom square for 50 ms.

Subsequently, the two white squares reappeared at the top and bottom of the screen for 1,500 ms. Participants used their right hand to indicate the location of the dot: pressing the “↑” key if it appeared at the top and the “↓” key if it appeared at the bottom. After this response, a prompt (“积极?消极?/Positive? Negative?”) appeared at the center of the screen for 3,000 ms, and participants used their left hand to judge the valence of the previously presented word, pressing “Z” for positive and “X” for negative. The assignment of response keys for positive and negative words was counterbalanced across participants, and the next trial began automatically after the response.

The experiment consisted of four blocks, each containing 42 trials. The first two trials of each block served as practice buffers and were excluded from analysis. The remaining 40 experimental trials included 10 words from each of the four categories. Each word appeared once in the top-key condition and once in the bottom-key condition, with block order counterbalanced across participants. Participants first completed a practice session to familiarize themselves with the task and response mapping before proceeding to the formal experiment, which lasted approximately 25 min.

### 3.3 Results and analysis

Two participants were excluded because their accuracy in the emotion word valence judgment task was below 75%. The remaining 22 participants achieved a mean valence judgment accuracy of 92.16% (SD = 8.65%). Only trials in which the valence judgment was correct were included in the analysis of the dot detection task. Trials with incorrect responses or reaction times (RTs) shorter than 200 ms were excluded, and extreme values were further removed using a ± 3 *SD* criterion. The overall data exclusion rate was less than 5%. Descriptive statistics for each condition are presented in Table 4.

**Table 4:** Mean reaction times and accuracy in the dot detection task under different lexical conditions in Experiment 2 (*M* ± SD).

Concreteness	Emotion valence	RTs (ms)		ACC (%)	
		Top	Bottom	Top	Bottom
Concrete	Positive	344 ± 72	383 ± 77	98.86 ± 2.15	99.77 ± 1.07
	Negative	361 ± 84	369 ± 88	99.32 ± 1.76	100.00 ± 0.00
Abstract	Positive	341 ± 72	387 ± 96	99.77 ± 1.07	99.09 ± 2.51
	Negative	360 ± 83	371 ± 73	99.77 ± 1.07	99.55 ± 1.47

The linear mixed-effects model revealed substantial variability in reaction times across participants, with the variance of participant intercepts estimated at 6585 (SD = 81). In contrast, the variance attributable to items was effectively zero, indicating negligible item-level variability. The residual variance was 9,265 (SD = 96), suggesting that while individual differences contributed to overall variability, the largest portion of variance remained unexplained by the model.

Analysis of the fixed effects revealed that the main effect of concreteness was not significant [*β* = 0.010, *t* = 0.180, *p* = 0.857, 95%*CI* = (−0.100, 0.120)]. The main effect of dot location was also not significant [*β* = −0.070, *t* = −0.617, *p* = 0.542, 95%*CI* = (−0.293, 0.153)]. The main effect of emotional valence approached significance [*β* = 0.107, *t* = 1.913, *p* = 0.056, 95%*CI* = (−0.003, 0.217)], suggesting a marginal tendency for reaction times to differ between positive and negative words.

Regarding the interaction effects, the interaction between emotional valence and dot location was significant [*β* = −0.250, *t* = −3.197, *p* = 0.001, 95%*CI* = (−0.403, −0.097)], indicating that the effect of emotional valence on reaction times depended on the spatial location of the dot. In contrast, the interaction between concreteness and emotional valence [*β* = 0.014, *t* = 0.183, *p* = 0.855, 95%*CI* = (−0.139, 0.168)] and the interaction between concreteness and dot location [*β* = −0.023, *t* = −0.289, *p* = 0.772, 95%*CI* = (−0.179, 0.133)] were not significant. Importantly, the three-way interaction among concreteness, emotional valence, and dot location was also not significant [*β* = −0.025, *t* = −0.227, *p* = 0.820, 95%*CI* = (−0.242, 0.192)].

Simple effects analyses revealed that when the dot appeared at the top, responses following positive words were faster than those following negative words (*t* = 3.670, *p* = 0.005). When the dot appeared at the bottom, responses following negative words were faster than those following positive words (*t* = −2.840, *p* = 0.006).

To further evaluate the absence of the three-way interaction, a Bayesian model comparison was conducted between the full model including the interaction and a reduced model excluding it. The results strongly favored the reduced model without the three-way interaction (BF₀₁ = 54.62), indicating that the data were substantially more likely under the model excluding the three-way interaction. However, given the limited statistical sensitivity for higher-order interactions in the present design, particularly in the absence of model-based power estimation for the linear mixed-effects models, these findings should be interpreted cautiously.

In Experiment 2, participants were required to judge the emotional valence of the words. A vertical spatial–valence congruency effect was observed. Importantly, lexical concreteness did not modulate this pattern: both concrete and abstract words showed similar congruency effects. These findings indicate that, for learners of Chinese as a foreign language, the spatial conceptual metaphor associated with Chinese emotion words appears to be relatively independent of lexical concreteness.

## 4 Discussion

This study investigated the influence of lexical concreteness on the embodied semantic representations of vocabulary in international learners of Chinese as a foreign language. Two experimental paradigms were employed: the spatial action Stroop task for verbs and the vertical spatial–valence congruency task for emotion words.

### 4.1 Embodied representations of foreign-language vocabulary in Chinese learners

Reaction time results indicate that learners of Chinese have developed embodied semantic representations for familiar foreign-language words, which manifest in both perceptual–motor simulations for verbs and spatial conceptual metaphors for emotion words (Vukovic and Williams, 2014; Xue et al., 2015; Zhang et al., 2020). Although previous studies have suggested that L2 proficiency may affect the strength or richness of embodied semantic representations (Ahlberg et al., 2018; Birba et al., 2020; Wang, 2019), participants in the present study were late bilinguals with limited Chinese proficiency, having passed only HSK Level 4. Despite this, they exhibited clear embodied effects when processing Chinese verbs and emotion words. This suggests that sufficiently high lexical familiarity enables foreign-language words to directly access embodied semantic representations.

Specifically, Experiment 1 required no explicit understanding of word meaning, yet participants still automatically engaged in semantically relevant perceptual–motor simulations, producing Stroop effects aligned with body movement directions. These results are consistent with previous findings (Dudschig et al., 2014; Shen et al., 2014; Xie et al., 2014, 2015), suggesting that the embodied effects observed during foreign-language comprehension may not arise from translating into the native language, but rather reflect a highly automatic association between the foreign language and perceptual–motor simulation. This implies that perceptual–motor simulation for foreign-language verbs is not a by-product of semantic comprehension (Birba et al., 2017; Vukovic et al., 2017). Instead, the observed compatibility effect suggests that perceptual–motor information can be automatically activated during verb processing and may contribute to the semantic representation of these words (Barsalou, 2010).

Experiment 2 employed the classical vertical spatial–valence congruency paradigm, demonstrating that emotion word semantics in learners of Chinese as a foreign language are associated with vertical spatial perception: positive words correspond to “up” and negative words to “down,” with semantic representations formed through metaphorical mappings to vertical space. Although some research suggests that conceptual metaphor representations exhibit language- and culture-specific characteristics (Hao and Fan, 2019), L2 learners did not show the horizontal spatial congruency effects commonly observed in English studies (Shen et al., 2014; Wu, 2019). Nevertheless, the vertical spatial–valence mappings of positive and negative emotion semantics appear to be relatively universal and stable.

### 4.2 Differences in embodied representation between concrete and abstract words

Across both experiments, concrete words exhibited richer and more diverse embodied representations, being represented and understood both through perceptual–motor simulation and metaphorical mappings to spatial perception. In contrast, abstract words were more limited, showing embodied representation only through vertical spatial conceptual metaphors. According to the multidimensional representation perspective, semantic representations arise from a combination of experiential and linguistic information (Bi, 2021; Ren et al., 2024). The representational characteristics of concrete and abstract words are determined by the types and proportions of associated internal and external experiences and linguistic information. Experiential information, derived from sensory perception, motor experiences, and life events, is richer and dominates the representation of concrete words, whereas abstract words rely primarily on emotional and linguistic information (Vigliocco et al., 2014). Compared with concrete words, abstract words depend more heavily on metaphorical mappings related to various perceptual experiences and linguistic associations, leading to differences in behavioral responses and neural activity (Barber et al., 2013).

### 4.3 Effects of concreteness on different embodied representation types

Regarding types of embodied semantic representation, concreteness influenced perceptual–motor simulation for verbs but did not affect vertical spatial conceptual metaphors for emotion words. Perceptual–motor simulations are sensitive to word concreteness: abstract words, lacking directly associated experiential information, are less able to engage in automatic semantic processing via perceptual–motor simulation. Conceptual metaphors, by contrast, map abstract semantic concepts onto perceptual experiences, using sensory–motor knowledge to represent and understand meaning. Such metaphorical mappings are not directly derived from concrete perceptual–motor experiences but are gradually constructed within specific linguistic and cultural contexts (Lakoff, 2008), exhibiting some degree of language and culture specificity. Therefore, as long as words carry clear emotional meaning, both concrete and abstract words produce the expected vertical spatial–valence congruency effect (positive–up, negative–down).

One possible explanation for the absence of a concreteness modulation concerns the nature of the experimental task. In Experiment 2, successful task performance required participants to process and maintain the emotional valence of the words. These task demands may have increased the salience of valence information, prompting participants to activate spatial representations associated with emotional valence. Consequently, the vertical space–valence congruency effect could have emerged relatively uniformly across different types of words, potentially attenuating observable differences between concrete and abstract items. In other words, the inherent emotional salience of the stimuli may have dominated participants’ processing, which could limit the detectability of concreteness effects. This suggests that the uniformity of the spatial congruency effect may reflect task-driven amplification of valence representations rather than equivalence in the underlying embodied semantic representation.

These findings provide a more nuanced perspective on embodied semantic representation in foreign-language learners. In Experiment 1, concrete verbs elicited perceptual–motor simulations even without explicit semantic classification, indicating that sensorimotor information can be automatically engaged during verb processing. Experiment 2 demonstrated a vertical spatial–valence congruency effect for emotion words regardless of concreteness, highlighting that emotional salience can drive spatial mapping even when lexical concreteness varies. Collectively, these results are consistent with a view of embodiment, where the degree and modality of perceptual–motor engagement depend on word type and task context.

By situating these results within prior research on L2 word processing and embodied cognition, the study advances our understanding of how concrete and abstract foreign-language words are represented differently and underscores the role of automatic sensorimotor activation in semantic comprehension. Future research could further explore the boundary conditions of these effects, for example by examining other word classes, incorporating cross-linguistic comparisons, or employing neuroimaging techniques to investigate the neural underpinnings of embodied representation.

Several aspects of the study limit the generalizability and interpretability of the findings. First, the absence of a native Chinese control group makes it difficult to determine whether the observed embodied effects reflect L2-specific processing or general Chinese lexical processing. Second, all participants shared a Vietnamese L1 background, so potential cross-linguistic transfer effects cannot be ruled out. Third, our study relied exclusively on behavioral measures; while the results are interpreted in terms of embodied semantic representations, no neural evidence was collected, and claims regarding neural grounding remain conceptual. Fourth, the statistical sensitivity of the present design remains uncertain. No model-based power analysis was conducted for the linear mixed-effects models, as such analyses typically require simulation-based approaches that depend on specifying uncertain parameters (e.g., variance components and random-effects structure). Consequently, the ability to detect higher-order interactions, particularly the critical three-way interactions, may be limited, and these effects should be interpreted as exploratory.

Future research could address these limitations by including native speakers as a control group, recruiting participants from diverse L1 backgrounds, incorporating neuroscientific methods to examine the neural basis of embodied semantic processing in foreign-language learners, and increasing sample sizes and the number of items to improve sensitivity to higher-order interactions.

## 5 Conclusion

This study examined the influence of lexical concreteness on embodied semantic representations in foreign-language Chinese learners through two experiments. The findings can be summarized as follows:

1. Only concrete foreign-language verbs automatically elicited perceptual–motor simulations; abstract verbs’ semantic representations did not depend on such simulations.
2. Vertical spatial conceptual metaphors for foreign-language emotion words were unaffected by lexical concreteness, with positive words mapping to “up” and negative words to “down.”
3. Concrete foreign-language words exhibited richer embodied representation, whereas abstract words’ embodied representation was limited to spatial conceptual metaphors.

These results demonstrate that concrete and abstract foreign-language words differ in their embodied representation, enriching embodied cognition theories of L2 semantic processing and providing empirical support for Chinese as a foreign language pedagogy. Foreign-language vocabulary instruction should consider word concreteness, leveraging perceptual–motor experiences and spatial conceptual metaphors to help learners develop robust embodied semantic representations and enhance vocabulary learning outcomes.

## Data Availability

The raw data supporting the conclusions of this article will be made available by the authors, without undue reservation.
