# Supplementary material for: The role of vocabulary concreteness in modulating embodied semantic representations in learners of Chinese as a foreign language
Source: Front Psychol. 2026 May 20;17:1791237. doi: 10.3389/fpsyg.2026.1791237 (PMC13230188; doi:10.3389/fpsyg.2026.1791237)
Supplement: Supplementary file 1 [file Table_1.DOCX]

Supplementary Material

# The materials used in the experiment

实验1使用的动词

| 具体趋近动词 | |  |  |  |  |  |  |  |  |
| --- | --- | --- | --- | --- | --- | --- | --- | --- | --- |
| 抓取 | 捕捉 | 采摘 | 插入 | 包扎 | 缝合 | 购买 | 喝茶 | 喝酒 | 拍手 |
| 拾取 | 收割 | 收集 | 收养 | 弯腰 | 咽下 |  |  |  |  |
| 具体远离动词 | |  |  |  |  |  |  |  |  |
| 搬家 | 剥落 | 播放 | 出售 | 丢弃 | 发球 | 发射 | 放牧 | 分发 | 寄出 |
| 离开 | 扔掉 | 射击 | 逃跑 | 逃走 | 投射 |  |  |  |  |
| 抽象趋近动词 | |  |  |  |  |  |  |  |  |
| 服从 | 引导 | 渴望 | 促使 | 吸收 | 积累 | 合作 | 接收 | 听信 | 领会 |
| 回顾 | 尝试 | 猎取 | 攻读 | 吸取 | 抚养 |  |  |  |  |
| 抽象远离动词 | |  |  |  |  |  |  |  |  |
| 传播 | 发布 | 反对 | 分解 | 告发 | 揭发 | 解放 | 扩大 | 扩张 | 散播 |
| 探索 | 推断 | 推广 | 消除 | 延迟 | 遗忘 |  |  |  |  |

实验2使用的情绪词

| 具体积极词 | |  |  |  |  |  |  |  |  |
| --- | --- | --- | --- | --- | --- | --- | --- | --- | --- |
| 伴侣 | 表扬 | 财产 | 飞翔 | 鼓掌 | 奖金 | 录取 | 旅行 | 母亲 | 朋友 |
| 微笑 | 舞蹈 | 宴会 | 拥抱 | 月亮 | 丈夫 | 整齐 | 祖国 | 冠军 | 庆祝 |
| 具体消极词 | |  |  |  |  |  |  |  |  |
| 爆炸 | 嘲笑 | 打架 | 发抖 | 罚款 | 疾病 | 禁止 | 开除 | 老鼠 | 批评 |
| 取消 | 去世 | 损坏 | 叹气 | 投降 | 拥挤 | 灾害 | 责备 | 战争 | 罪犯 |
| 抽象积极词 | |  |  |  |  |  |  |  |  |
| 风趣 | 光辉 | 吉祥 | 杰出 | 精彩 | 乐观 | 前景 | 前途 | 神奇 | 审美 |
| 天堂 | 伟大 | 新颖 | 信誉 | 雄伟 | 优点 | 优秀 | 友谊 | 真理 | 正义 |
| 抽象消极词 | |  |  |  |  |  |  |  |  |
| 悲观 | 别扭 | 草率 | 风险 | 狠心 | 忽视 | 荒唐 | 混乱 | 嫉妒 | 假装 |
| 紧迫 | 茫然 | 难堪 | 玩弄 | 消极 | 压力 | 压制 | 野心 | 犹豫 | 自卑 |

# Materials used in the experiment - English translation version

Verbs Used in Experiment 1

| Concrete approach verbs | | |  |  |  |  |  |  |  |
| --- | --- | --- | --- | --- | --- | --- | --- | --- | --- |
| grasp | catch | pick | insert | bandage | sew | purchase | drink tea | drink alcohol | clap |
| collect | harvest | gather | adopt | bend | swallow |  |  |  |  |
| Concrete avoidance verbs | | |  |  |  |  |  |  |  |
| move | peel | play (media) | sell | discard | serve (ball) | launch | herd | distribute | mail |
| leave | throw | shoot | flee | escape | project |  |  |  |  |
| Abstract approach verbs | | |  |  |  |  |  |  |  |
| obey | guide | desire | motivate | absorb | accumulate | cooperate | receive | heed | comprehend |
| review | attempt | hunt | pursue | assimilate | foster |  |  |  |  |
| Abstract avoidance verbs | | |  |  |  |  |  |  |  |
| spread | publish | oppose | decompose | report | expose | liberate | expand | enlarge | disseminate |
| explore | infer | promote | eliminate | delay | forget |  |  |  |  |

Emotion Words Used in Experiment 2

| Concrete Positive Words | | |  |  |  |  |  |  |  |
| --- | --- | --- | --- | --- | --- | --- | --- | --- | --- |
| partner | praise | property | flying | clap | bonus | admission | travel | mother | friend |
| smile | dance | banquet | hug | moon | husband | neatness | homeland | champion | celebration |
| Concrete Negative Words | | |  |  |  |  |  |  |  |
| explosion | ridicule | fight | shiver | fine | disease | prohibition | dismissal | rat | criticism |
| cancellation | death | damage | sigh | surrender | crowding | disaster | blame | war | criminal |
| Abstract Positive Words | | |  |  |  |  |  |  |  |
| humorous | brilliant | auspicious | outstanding | splendid | optimistic | prospects | future | magical | aesthetic |
| paradise | great | novel | reputation | majestic | merit | excellent | friendship | truth | justice |
| Abstract Negative Words | | |  |  |  |  |  |  |  |
| pessimistic | awkward | hasty | risk | cruel | neglect | absurd | chaotic | jealousy | pretend |
| urgent | confused | embarrassed | manipulate | negative | stress | suppress | ambition | hesitate | inferior |
